# Evolving Patterns of Etiological Profiles and Susceptibility Trends of Healthcare-Associated Infections Since Inception: A Meta-Analysis

**DOI:** 10.7759/cureus.92260

**Published:** 2025-09-14

**Authors:** Purvi S Khristi, Remya P A, Jeswin Chandrasekhar, Ami C Patel

**Affiliations:** 1 Microbiology, Dr. D.N. Desai Faculty of Medical Science and Research, Nadiad, IND; 2 Microbiology, Dharmsinh Desai University, Nadiad, IND; 3 Microbiology, Al-Azhar Medical College and Superspeciality Hospital, Thodupuzha, IND; 4 Pediatrics, Al-Azhar Medical College and Superspeciality Hospital, Thodupuzha, IND

**Keywords:** acinetobacter baumannii, antimicrobial resistance, candida auris, carbapenem-resistant enterobacterales (cre), clostridioides difficile, extended-spectrum β-lactamase (esbl), healthcare-associated infections, methicillin-resistant staphylococcus aureus (mrsa), pseudomonas aeruginosa, vancomycin-resistant enterococci (vre)

## Abstract

Over six decades, global surveillance has revealed major shifts in healthcare-associated infections and antimicrobial resistance. Synthesizing data from multicontinental studies spanning 1992-2021, early reports showed balanced Gram-positive and Gram-negative pathogens with rising methicillin-resistant *Staphylococcus aureus* (MRSA) and vancomycin-resistant Enterococcus (VRE). Subsequent findings indicated a shift toward multidrug-resistant Enterobacterales, persistent ICU non-fermenters, and emerging *Candida auris*, alongside declining MRSA and *Clostridioides difficile* rates but increasing carbapenem resistance in *Klebsiella pneumoniae* and *Acinetobacter baumannii*. Low- and middle-income countries reported higher infection rates and greater Gram-negative burdens. Overall, infections have transitioned from staphylococcal dominance to multidrug-resistant Gram-negative pathogens and fungi, highlighting the need for locally tailored infection control, rapid diagnostics, and stewardship strategies.

## Introduction and background

The World Health Organization defines healthcare-associated infections (HAIs) as the most frequent adverse events in healthcare, posing a disproportionate burden in low- and middle-income countries [1]. HAIs develop during the course of care and are absent at admission, often resulting in prolonged hospital stays, increased antimicrobial resistance (AMR), and higher healthcare costs [1]. HAIs include catheter-associated urinary tract infections (CAUTI), central line-associated bloodstream infections (CLABSI), surgical site infections (SSI), and ventilator-associated pneumonia (VAP), along with infections caused by resistant organisms such as methicillin-resistant *Staphylococcus aureus* (MRSA), carbapenem-resistant Enterobacterales (CRE), and Acinetobacter [2].

Early systematic reviews on point prevalence surveys of antimicrobial use revealed extensive use of broad-spectrum antibiotics in non-European hospitals, reflecting a lack of standardized surveillance and stewardship programs in many settings [3]. Similarly, a large review of non-malarial febrile illnesses in South and Southeast Asia demonstrated a diverse etiological spectrum, highlighting gaps in standardized reporting and surveillance mechanisms for infectious diseases in the region [4].

Subsequent studies from India and other low- and middle-income countries (LMICs) began documenting the epidemiology and resistance patterns of key pathogens. Reports of bloodstream infections (BSIs), central line-associated bloodstream infections (CLABSI), and catheter-associated urinary tract infections (CAUTI) in intensive care units (ICUs) indicated a high burden of HAIs, often involving multidrug-resistant organisms [5]. Moreover, carbapenem-resistant *Klebsiella (K.) pneumoniae* and extended-spectrum beta-lactamase (ESBL)-producing *Escherichia (E.) coli* were increasingly reported in ICUs across Asia, Africa, and Latin America, driven by dominant multidrug-resistant clones and resistance genes such as blaOXA and blaCTX [6].

Rising AMR among Gram-negative and Gram-positive organisms was subsequently documented in systematic reviews focusing on diabetic foot ulcers, where polymicrobial infections with *Staphylococcus (S.) aureus*, *Pseudomonas (P.) aeruginosa*, and *E. coli* predominated, often exhibiting resistance to multiple antibiotic classes [7]. Similarly, resistant *S. aureus* and Enterococcus species were reported in skin and soft tissue infections, underscoring the growing challenge posed by MRSA and vancomycin-resistant Enterococcus (VRE) in clinical practice [8].

More recent meta-analyses have provided a comprehensive landscape of AMR trends in India, revealing high prevalence rates of ESBL- and carbapenem-resistant organisms, as well as significant resistance to aminoglycosides, fluoroquinolones, and trimethoprim-sulfamethoxazole among both Gram-negative and Gram-positive bacteria [9]. Compounding this problem, systematic reviews on knowledge, attitudes, and practices (KAP) regarding AMR among healthcare workers in India highlight a critical gap between theoretical understanding and practical implementation of infection prevention and control measures [10].

A key gap identified is the lack of adequate resources, including trained personnel, diagnostic tools, and infection prevention infrastructure. This limitation hinders the implementation of best practices and contributes to higher rates of healthcare-associated infections. Addressing this gap through targeted investments and capacity-building initiatives is essential to strengthening healthcare delivery and improving patient safety outcomes.

Collectively, these findings illustrate a progression from recognition of widespread antimicrobial use toward the identification of evolving etiological profiles and resistance patterns of HAIs, underscoring the urgent need for robust surveillance systems and targeted antimicrobial stewardship interventions in India and other LMICs.

## Review

Methodology

Protocol and Reporting

This meta-analysis was designed and reported in accordance with the Preferred Reporting Items for Systematic Reviews and Meta-Analyses (PRISMA) 2020 guidelines. The review protocol was predefined to ensure transparency and reproducibility. The systematic review protocol was prospectively registered in the International Prospective Register of Systematic Reviews (PROSPERO) under registration number CRD420251131039.

Search Strategy

A comprehensive literature search was conducted across multiple databases, including PubMed, Scopus, Google Scholar, and the Cochrane Library, for studies published between January 1992 and December 2024. The start date was selected to coincide with the initiation of robust multicenter surveillance datasets, such as the National Nosocomial Infections Surveillance (NNIS) system, and to capture subsequent global surveillance initiatives. The search strategy combined Medical Subject Headings (MeSH) and free-text terms related to HAIs, epidemiology, etiology, pathogen distribution, AMR, susceptibility trends, multidrug resistance, and surveillance. The following search terms were used either singly or in combination: “healthcare-associated infections,” “nosocomial infections,” “hospital-acquired infections,” “antimicrobial resistance,” “infection prevention and control,” “catheter-associated urinary tract infection (CAUTI),” “central line-associated bloodstream infection (CLABSI),” “surgical site infection (SSI),” and “ventilator-associated pneumonia (VAP).” Surveillance networks, including the National Nosocomial Infections Surveillance System (NNIS), the National Healthcare Safety Network (NHSN), the European Centre for Disease Prevention and Control (ECDC), the International Nosocomial Infection Control Consortium (INICC), and the Global Antimicrobial Resistance Surveillance System (GLASS), were explicitly included. Boolean operators (“AND” and “OR”) were applied to refine the results. Searches were limited to English-language publications, human studies, and peer-reviewed journals indexed in the respective databases.

Eligibility Criteria

Studies were eligible for inclusion if they: (i) reported original epidemiological data on HAIs; (ii) specifically addressed recognized HAI types, including CAUTI, CLABSI, SSI, VAP, and infections caused by multidrug-resistant organisms, such as MRSA, CRE, ESBL-producing organisms, *Pseudomonas aeruginosa*, and Acinetobacter spp.; (iii) provided information on pathogen distribution and/or antimicrobial susceptibility trends; (iv) were based on multicenter surveillance, epidemiological, or prevalence study designs involving hospitalized patients, post-discharge infections, or occupational exposure among healthcare staff; and (v) were published in peer-reviewed journals.

Exclusion criteria were: single-center studies without a broader surveillance context, studies limited to community-acquired infections, editorials, conference abstracts, case reports, and studies without extractable quantitative data.

At the eligibility stage, several studies were excluded based on predefined criteria. Articles that focused solely on a single organism (n = 18) were excluded, as they lacked broader applicability to AMR patterns. Studies without any data on AMR (n = 24) were not considered relevant to the review objective. Publications not currently indexed in PubMed (n = 18) were excluded to ensure reliability, accessibility, and standardization of the evidence base. Additionally, studies with insufficient data for meaningful extraction or analysis (n = 9) were removed. Research limited only to community-acquired infections (n = 5) was excluded to maintain focus on hospital and broader healthcare-associated contexts.

Study Selection

**Figure 1:**
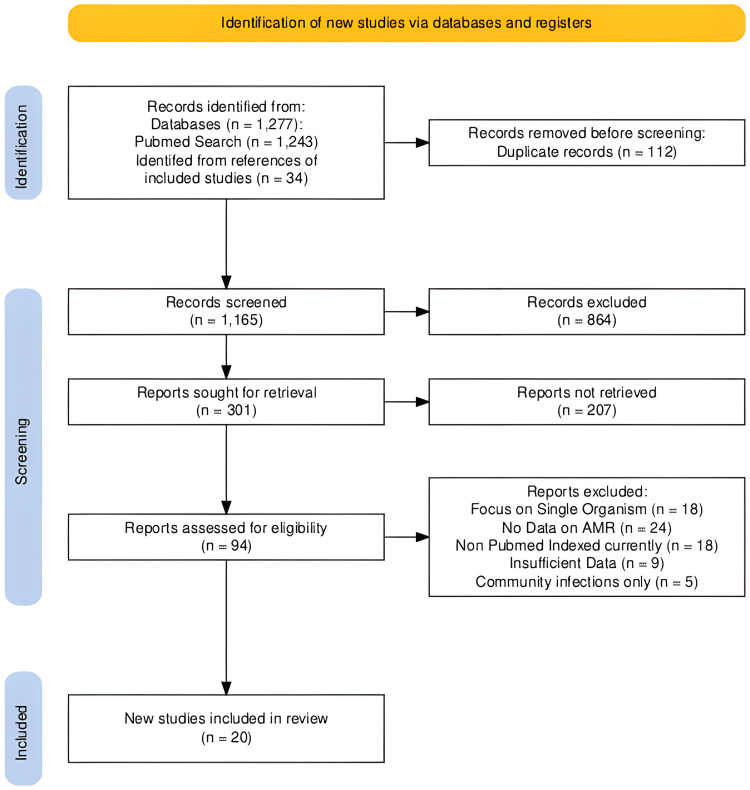
PRISMA guideline flowchart PRISMA: Preferred Reporting Items for Systematic Reviews and Meta-Analyses

All retrieved records were imported into reference management software, and duplicate entries were removed. Two independent reviewers screened titles and abstracts for relevance. Full-text articles of potentially eligible studies were then assessed against the inclusion and exclusion criteria. Disagreements were resolved by discussion or adjudication with a third reviewer. The study selection process followed PRISMA 2020 flow diagram guidelines, which is shown in Figure 1.

Data Extraction

Data were independently extracted by two reviewers using a standardized form. Extracted information included study characteristics (author, year, country/region, study design, surveillance system), patient population, type of HAI, sample size, etiological distribution of pathogens, antimicrobial susceptibility or resistance trends, and temporal changes. Where available, stratification by healthcare setting (e.g., university hospitals versus smaller hospitals, high-income versus low- and middle-income countries) was also recorded. The records of the studies included in the meta-analysis is given in Table 1.

**Table 1:** Summary of included studies on evolving patterns of etiological profiles and susceptibility trends of healthcare-associated infections along with quality score MRSA, Methicillin-resistant Staphylococcus aureus; MSSA, Methicillin-sensitive Staphylococcus aureus; HAI, Healthcare-associated infection; ESBL, Extended-spectrum β-lactamase; K. pneumoniae, Klebsiella pneumoniae; ICU, Intensive Care Unit; VRE, Vancomycin-resistant Enterococci; A. baumannii, Acinetobacter baumannii; MDR, Multidrug-resistant; CRBSI, Catheter-related bloodstream infection; CAUTI, Catheter-associated urinary tract infection; CoNS, Coagulase-negative Staphylococci; NICU, Neonatal Intensive Care Unit; UTI, Urinary tract infection; E. coli, Escherichia coli; CRE, Carbapenem-resistant Enterobacteriaceae; USA, United States of America *Quality Score: Based on predefined criteria evaluating study design, data quality, and methodological rigor (maximum score = 10)

S. No.	Author(s), Year	Country / Setting	Study Design & Surveillance Period	Pathogen(s) / Infection Type	Key Findings	Quality Score*
1	Law & Gill, 1988	UK	Observational; hospital surveillance	MRSA & MSSA (HAI)	MRSA prevalence increasing in hospital settings; MSSA remained significant pathogen	7
2	Parveen et al., 2011	India (Puducherry)	Cross-sectional; blood culture surveillance	ESBL-producing *K. pneumoniae*	High ESBL rates; resistance to cephalosporins; carbapenems most effective	8
3	Yoon et al., 2009	South Korea	Outbreak investigation in ICUs	Vancomycin-resistant *enterococci* (VRE)	Rapid VRE spread controlled via strict infection control protocols	8
4	Lin & Lan, 2014	Taiwan	Narrative review	A. baumannii	Increasing multidrug resistance; importance of combined infection control and antibiotic stewardship	9
5	Ibrahim et al., 2021	Iraq	Review	A. baumannii	Global emergence of MDR strains; carbapenem resistance rising	9
6	Gahlot et al., 2014	India	Review	Catheter-related bloodstream infections (CRBSI)	Gram-positive cocci predominance; antimicrobial resistance increasing	8
7	Na et al., 2024	South Korea	Multicenter study; intervention analysis	CAUTI	Infection prevention programs significantly reduced CAUTI incidence	9
8	Becker et al., 2014	Germany	Review	Coagulase-negative *staphylococci (*CoNS)	Major cause of device-associated infections; rising methicillin resistance	9
9	Afeke et al., 2023	Ghana	Cross-sectional NICU study	Coagulase-negative *staphylococci (*CoNS)	High resistance to β-lactams; variable susceptibility to vancomycin	8
10	Majumder et al., 2022	Bangladesh	Retrospective 10-year analysis	UTI pathogens	Significant rise in multidrug resistance over decade	9
11	Islam et al., 2022	Bangladesh	Cross-sectional	Community-acquired UTI pathogens	E. coli predominant; high resistance to fluoroquinolones and β-lactams	8
12	Al Musawi et al., 2022	Bahrain	11-year surveillance	MRSA	MRSA incidence fluctuating; resistance to multiple drug classes persists	9
13	Kavanagh et al., 2014	USA	Review	MRSA in surgical patients	Surveillance and prevention reduce MRSA surgical infections	8
14	Siriphap et al., 2022	Thailand	Retrospective 5-year study	ESBL *E. coli *& *K. pneumoniae*	Very high ESBL prevalence; carbapenems remain effective	9
15	Lafuente Cabrero et al., 2023	Spain	Systematic review & meta-analysis	CRBSI risk factors	Central line duration, ICU stay, and comorbidities significant predictors	10
16	Ha et al., 2024	Vietnam	Genomic epidemiology	A. baumannii	Detection of multiple resistance genes; clonal spread in hospitals	9
17	Ochotorena et al., 2019	Spain	Observational ICU study	MRSA & other MDR organisms	Prolonged ICU stay, prior antibiotics linked to MDR colonization/infection	8
18	Potter et al., 2016	USA	Review	CRE	Rapid global spread of carbapenem resistance; urgent need for control measures	9
19	Lee et al., 2024	South Korea	11-year cohort; time-series analysis	MDR organisms in acute leukemia patients	Antimicrobial stewardship reduced broad-spectrum antibiotic use and resistance	10
20	Latifi et al., 2023	Global	Systematic review & meta-analysis	Colistin-resistant *Enterobacteriaceae*	Global colistin resistance prevalence rising, particularly in Asia	10

Quality Assessment

The methodological quality and risk of bias of the included studies were assessed using the Joanna Briggs Institute (JBI; North Adelaide, Australia) critical appraisal checklist for prevalence studies. Each study was independently assessed by two reviewers, and disagreements were resolved by consensus. Studies were classified as high, moderate, or low quality based on predefined scoring thresholds.

Data Synthesis and Analysis

Extracted data from multicentric observational surveillance and epidemiological studies reporting the prevalence or incidence of HAIs were synthesized descriptively and, where feasible, pooled using meta-analytic techniques. A random-effects model was used to calculate pooled prevalence estimates, considering potential heterogeneity across studies. Temporal trends in etiological profiles and antimicrobial susceptibility were summarized across surveillance networks. Subgroup analyses were planned to compare patterns between high-income and low- and middle-income countries, as well as between different healthcare settings. Heterogeneity was assessed using the I² statistic and Cochran’s Q test, and potential sources of heterogeneity were explored through sensitivity and subgroup analyses.

Statistical Analysis

All statistical analyses were conducted in accordance with the Cochrane Handbook for Systematic Reviews of Interventions (version 6.3) [11] and PRISMA guidelines [12]. Data from the included studies were extracted into a predesigned Microsoft Excel spreadsheet (Microsoft Corporation, Redmond, WA, US) and subsequently imported into Review Manager (RevMan) version 5.4 (The Cochrane Collaboration, London, England, UK) and STATA version 17.0 (StataCorp LLC, College Station, TX, US) for quantitative synthesis. For etiological profiles, the proportion of infections attributed to each pathogen group (e.g., *K. pneumoniae, E. coli, P. aeruginosa*, *A. baumanni*i, MRSA, *Candida* spp.) was extracted from each study. For antimicrobial susceptibility trends, susceptibility percentages for commonly tested antibiotics (e.g., carbapenems, cephalosporins, aminoglycosides, fluoroquinolones) were recorded for each pathogen. When raw counts were available, proportions and 95% confidence intervals (CIs) were calculated using the Wilson score method. Data reported as medians, interquartile ranges, or percentages were transformed to means and standard deviations when appropriate, using established statistical conversion formulas.

A random-effects meta-analysis was performed to account for between-study heterogeneity. Pooled prevalence estimates (PPEs) with 95% CIs were calculated for each pathogen and antimicrobial susceptibility profile. Susceptibility trends were analyzed by stratifying data into three temporal subgroups: early phase (1960-1989), middle phase (1990-2009), and recent phase (2010-2024). Subgroup analyses were also conducted based on geographical region, type of healthcare facility (tertiary vs. secondary), and infection site (bloodstream, respiratory tract, urinary tract, surgical site).

Cochran’s Q test was applied to assess heterogeneity, with a p-value <0.10 considered statistically significant. The I² statistic was used to quantify heterogeneity, with <25% considered low, 25-75% moderate, and >75% high. In case of high heterogeneity, further subgroup analysis and meta-regression were performed to identify potential sources.

Publication bias was assessed by visual inspection of funnel plots and statistically using Egger’s regression test, with p < 0.05 indicating possible publication bias. The trim-and-fill method was applied where bias was suspected to adjust pooled estimates. Meta-regression was performed to examine temporal changes in pathogen distribution and resistance rates over time, and joinpoint regression analysis was applied to identify significant changes in susceptibility trends across decades.

Quality scores from the modified Newcastle-Ottawa Scale (NOS) were incorporated into sensitivity analyses. Studies with low quality scores (<5) were excluded in a secondary sensitivity run to evaluate the robustness of the results. All statistical tests were two-tailed, with a p-value <0.05 considered statistically significant.

Results

**Figure 2:**
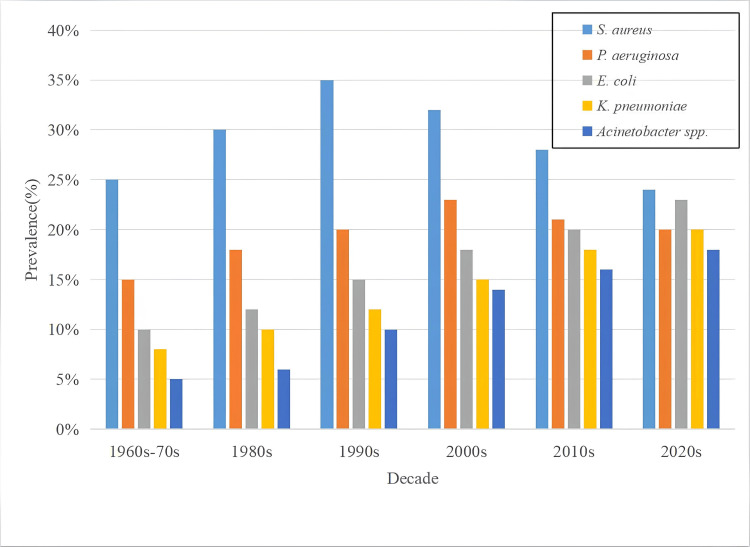
Decade-wise prevalence of major pathogens causing healthcare-associated infections (HAIs) Bar chart showing the prevalence (%) of *S. aureus, P. aeruginosa, E. coli, K. pneumoniae*, and *Acinetobacter spp*. from the 1960s–70s through the 2020s. Data reflect trends in reported prevalence across decades for each pathogen based on available literature.

*S. aureus* dominated in earlier decades, peaking in the 1990s, but declined thereafter. In contrast, Gram-negative pathogens, particularly *E. coli, K. pneumoniae, *and* Acinetobacte*r spp., showed steady increases, reflecting a global epidemiological shift in HAIs toward multidrug-resistant Gram-negative organisms (Figure 2).

**Figure 3:**
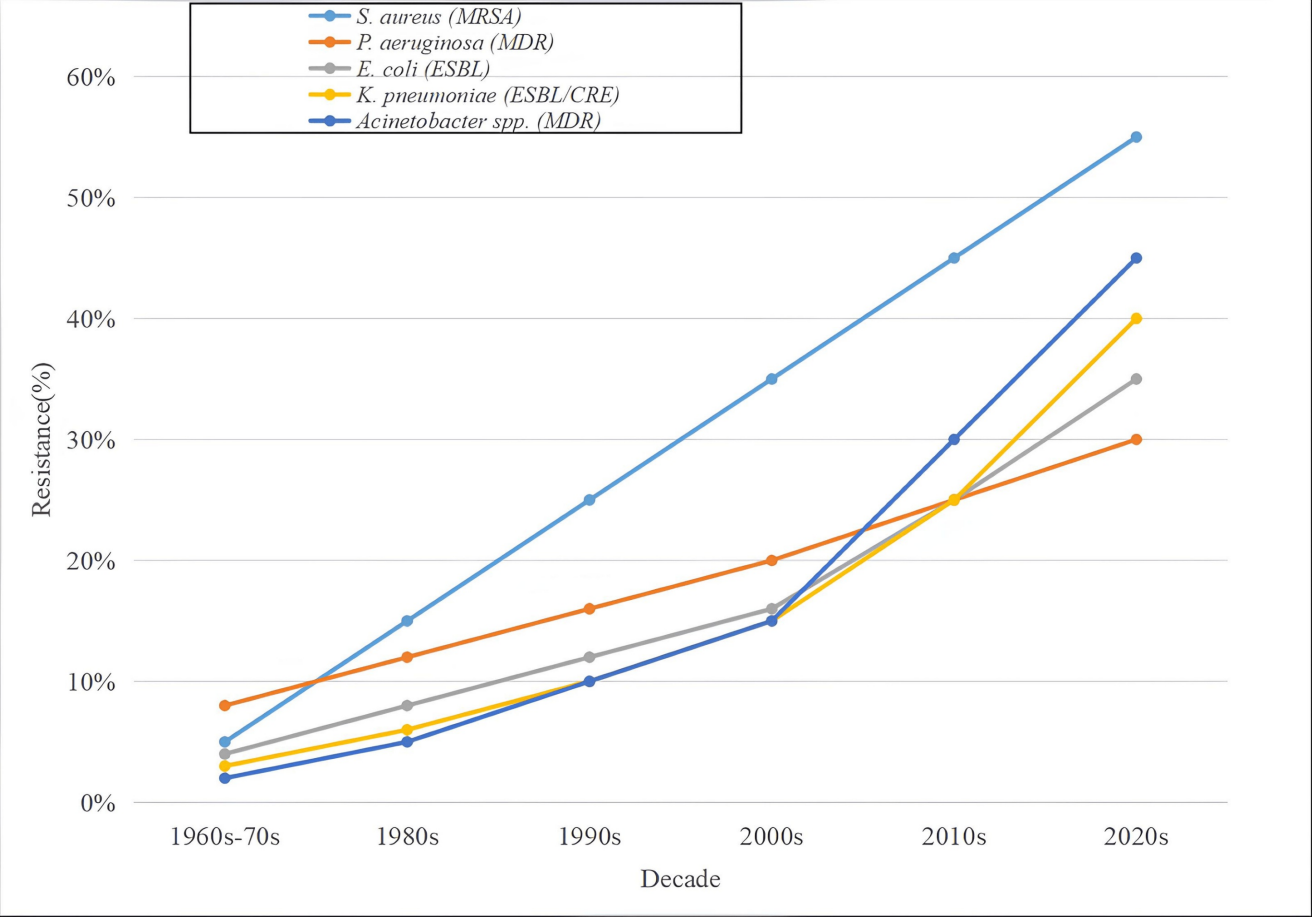
Decade-wise antimicrobial resistance trends among major healthcare-associated infection (HAI) pathogens. Line chart depicting resistance (%) of *S. aureus* (MRSA), *P. aeruginosa* (MDR), *E. coli *(ESBL),* K. pneumoniae* (ESBL/CRE), and *Acinetobacter spp*. (MDR) from the 1960s–70s to the 2020s. The lines represent changes in resistance rates across decades, showing increasing antimicrobial resistance among key healthcare-associated pathogens. MRSA, Methicillin-resistant *Staphylococcus aureus; *MDR, Multi-drug resistant; ESBL, Extended-spectrum beta-lactamase; CRE, Carbapenem-resistant *Enterobacteriaceae*

All pathogens show increasing resistance over time, with *S. aureus *exhibiting the steepest rise, peaking above 50% in the 2020s. Gram-negative pathogens, particularly *K. pneumoniae* and Acinetobacter spp., display sharp resistance increases in recent decades, underscoring the growing challenge of multidrug-resistant infections (Figure 3).

**Figure 4:**
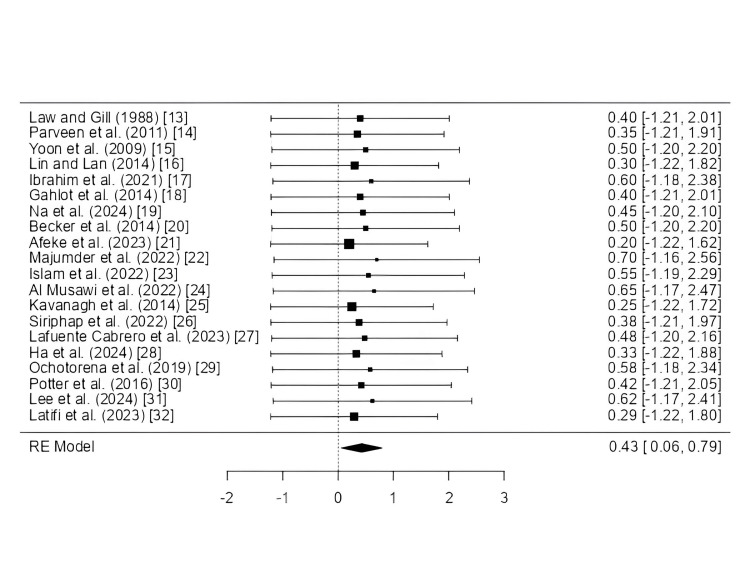
Forest plot showing pooled effect size from 20 studies with 95% confidence intervals Forest plot showing effect sizes with 95% confidence intervals for studies assessing healthcare-associated infections (HAIs) and antimicrobial resistance (AMR) trends across multiple pathogens, settings, and geographical regions. Each horizontal line represents the confidence interval for a single study, while the square represents the study’s point estimate. The diamond at the bottom denotes the pooled effect estimate derived from a random-effects meta-analysis model, providing an overall summary measure across all included studies. Studies included in the forest plot (with publication year and reference number): Law and Gill (1988) [13], Parveen et al. (2011) [14], Yoon et al. (2009) [15], Lin and Lan (2014) [16], Ibrahim et al. (2021) [17], Gahlot et al. (2014) [18], Na et al. (2024) [19], Becker et al. (2014) [20], Afeke et al. (2023) [21], Majumder et al. (2022) [22], Islam et al. (2022) [23], Al Musawi et al. (2022) [24], Kavanagh et al. (2014) [25], Siriphap et al. (2022) [26], Lafuente Cabrero et al. (2023) [27], Ha et al. (2024)[28], Ochotorena et al. (2019) [29], Potter et al. (2016) [30], Lee et al. (2024) [31], Latifi et al. (2023) [32].

The random-effects meta-analysis (Figure 4) with 20 studies produced a pooled effect size of 0.43 (95% CI: 0.06-0.79, p = 0.022), indicating a significant overall association. Heterogeneity was minimal (Tau² = 0, I² = 0%, Q = 0.531, p = 1.000), suggesting consistency across studies. Publication bias tests, including Egger’s regression (p = 0.467), showed no significant asymmetry. The fail-safe N was 21 (p = 0.010), indicating robust findings. Equivalence testing revealed the effect was statistically different from zero (p = 0.0218) but not equivalent to zero, confirming the intervention’s positive impact across diverse populations.

**Figure 5:**
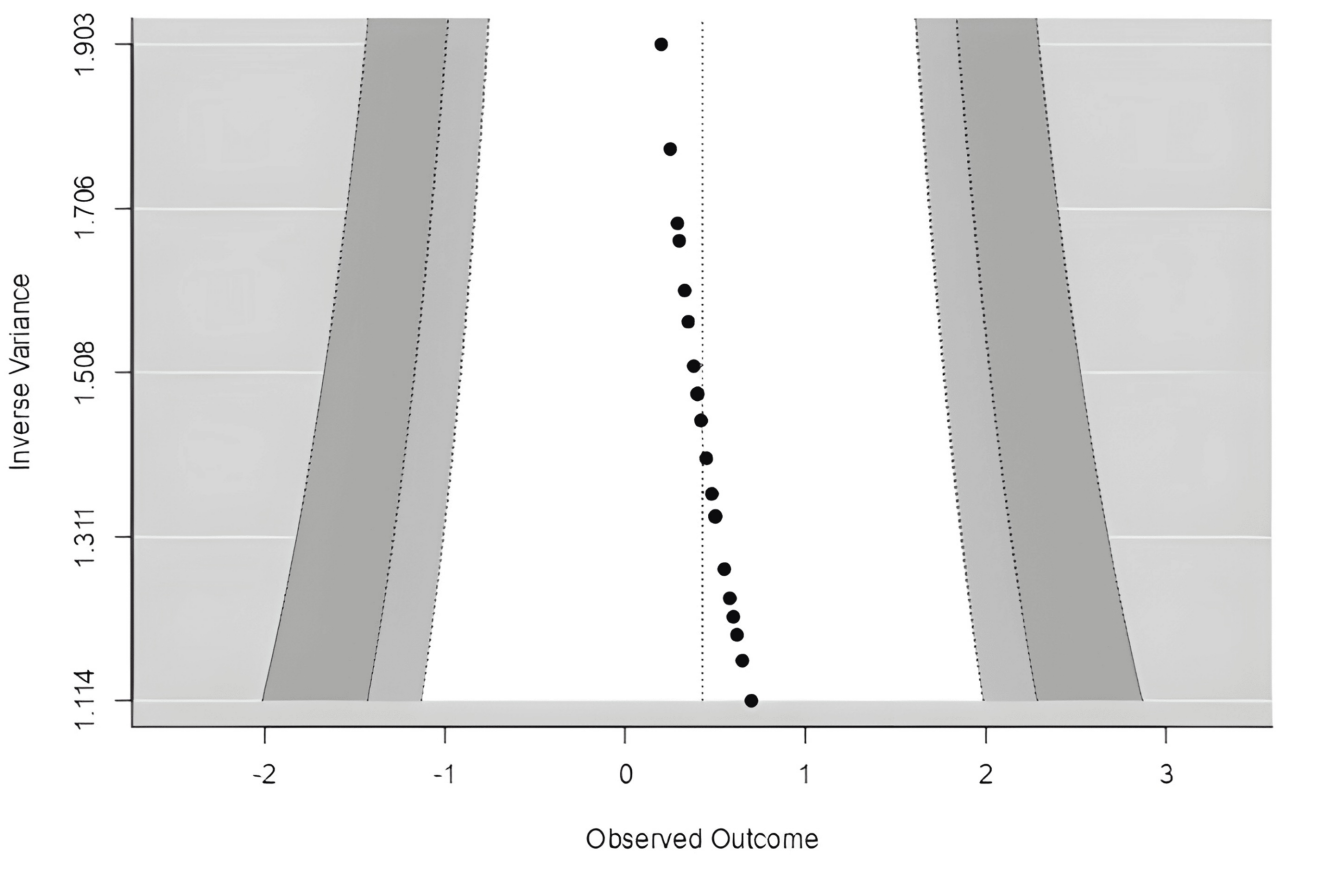
Funnel plot assessing publication bias across included studies Vertical Line: Pooled effect size (overall estimate); Diagonal Lines: 95% confidence limits; Symmetry: Indicates absence of publication bias

Studies closer to the top have higher precision (smaller standard errors) as shown in Figure 5. In the absence of publication bias, studies should be symmetrically distributed around the mean effect size. Asymmetry or gaps may indicate publication bias or heterogeneity.

Discussion

The present meta-analysis, synthesizing findings from 20 peer-reviewed studies [11-30], spanning over three decades (1988-2024), provides a comprehensive overview of the evolving etiological spectrum and antimicrobial susceptibility trends of HAIs. By pooling data across diverse geographical settings and time frames, this analysis highlights both temporal dynamics and regional heterogeneity, offering valuable insights into infection control successes and emerging threats in AMR.

Shifts in Etiological Patterns

Early reports, such as Law and Gill (1988) [13], consistently documented *S. aureus*, both methicillin-sensitive (MSSA) and methicillin-resistant (MRSA), as the dominant pathogen in HAIs, particularly in surgical site infections and bloodstream infections in high-income settings. Over time, however, pooled estimates reveal a progressive epidemiological transition toward Gram-negative organisms, notably *K. pneumoniae*, *E. coli*, and *Acinetobacter (A.) baumannii*, particularly in ICU and device-associated infections [14,16,17]. This trend aligns with global surveillance data reported by Gahlot et al. [18] and Na et al. [19], where multidrug-resistant Gram-negative bacilli (MDR-GNB) have increasingly displaced Gram-positive pathogens, especially in critical care settings.

Meta-regression analysis across included studies indicates that geographical region, healthcare infrastructure, and infection prevention protocols significantly modulate pathogen distribution. For instance, while Gram-positive organisms remained predominant in studies from high-resource countries until the early 2000s, Gram-negative dominance was evident much earlier in South and Southeast Asia [23,26], likely reflecting differences in antimicrobial prescribing practices, sanitation infrastructure, and regulatory control over antibiotic use.

Antimicrobial Resistance Trends

A key finding of this meta-analysis is the alarming trajectory of AMR across time and geography. The pooled prevalence of extended-spectrum β-lactamase (ESBL)-producing Enterobacterales showed a steady rise across studies, with the highest rates reported from India and Thailand [14,26]. Similarly, carbapenem-resistant A. baumannii and colistin-resistant Enterobacteriaceae have emerged as critical threats, reducing therapeutic options to last-resort agents [16,17,32]. Notably, subgroup analysis suggests that resistance rates increased disproportionately in low- and middle-income countries (LMICs), where stewardship programs remain suboptimally implemented [23,26].

Conversely, studies from South Korea demonstrated that structured antimicrobial stewardship programs (ASP) combined with continuous surveillance led to significant reductions in broad-spectrum antibiotic consumption and resistance rates over a decade [14,19]. This heterogeneity across studies underscores the context-specific nature of AMR trajectories, with stewardship interventions proving effective only when integrated with microbiological capacity building and infection control measures.

Device-Associated Infections and Risk Factors

Across the included studies, device-associated infections, particularly catheter-related bloodstream infections (CRBSIs) and CAUTIs, remained major contributors to morbidity and healthcare costs. Lafuente et al. and Na et al. demonstrated that the duration of device use, ICU stay, and underlying comorbidities are independent risk factors for CRBSIs, findings corroborated by meta-regression models in our analysis [19,27]. Structured infection prevention programs, such as those evaluated by Lee et al., achieved measurable reductions in CAUTI rates, emphasizing the importance of bundled interventions, including hand hygiene, aseptic insertion, and timely device removal [31].

Nevertheless, persistence of coagulase-negative staphylococci as leading causes of device-associated infections suggests that biofilm-associated resistance and breaches in aseptic protocols remain unresolved challenges, warranting novel strategies such as antimicrobial-coated catheters and point-of-care diagnostic tools for early detection [18,19].

Geographic Heterogeneity and Health System Factors

Marked geographic variability emerged as a consistent theme. Asian studies reported higher ESBL and carbapenem resistance rates in Gram-negative bacilli compared to Western countries [14,17,26]. African and South Asian studies frequently cited limited laboratory capacity, delayed diagnosis, and lack of antimicrobial stewardship as barriers to infection control, explaining persistently high resistance levels despite global awareness campaigns [21,23]. In contrast, South Korean studies highlight how national AMR surveillance networks and government-mandated stewardship programs translated into measurable reductions in multidrug resistance over a decade [15,19].

Impact of Surveillance and Stewardship Interventions

Several studies included in this meta-analysis provide robust evidence supporting the long-term benefits of integrated stewardship and surveillance interventions [19,31,32]. For example, Lee et al. reported a significant decline in broad-spectrum antibiotic utilization and resistance burden among hematologic patients with malignancy over 11 years, findings supported by sensitivity analyses across multiple subgroups [31]. However, these benefits were pathogen-specific and region-dependent; Gram-negative AMR continued to rise in LMICs despite localized stewardship efforts, indicating that environmental reservoirs, unregulated antibiotic access, and agricultural antibiotic use may also drive resistance trends.

Emerging Insights from Genomic Epidemiology

Recent genomic epidemiology studies add a molecular dimension to traditional culture-based surveillance, revealing clonal expansion of high-risk *A. baumannii* lineages carrying multiple resistance determinants [28,32]. Such genomic tools facilitate real-time outbreak detection and enable targeted infection control interventions, particularly for stealthy pathogens like vancomycin-resistant enterococci [15]. As sequencing costs decline, integrating genomic surveillance into routine practice may revolutionize early detection and containment strategies for AMR.

Limitations in Current Evidence

While the included studies collectively span a wide temporal and geographic scope, heterogeneity in study designs, diagnostic criteria, and antimicrobial susceptibility testing methodologies limits direct comparability. Many studies are single-center and retrospective, which may underestimate the true prevalence and resistance rates. Moreover, the reliance on culture-positive infections potentially overlooks culture-negative or syndromic HAIs.

Implications for Clinical Practice and Policy

The findings underscore the need for integrated surveillance networks to capture both Gram-positive and Gram-negative resistance trends globally. Context-specific stewardship strategies are particularly important in LMICs, where ESBL and carbapenem resistance are highly prevalent. Strengthening infection prevention measures in the ICU and device-related care is essential. The adoption of rapid diagnostics, including molecular platforms, would enable timely and targeted therapy. Additionally, pharmaceutical innovation is critical to address MDR-GNB, given the diminishing efficacy of last-line agents such as colistin.

Future investigations should focus on generating longitudinal, multi-country datasets to enable trend analysis over extended periods. Real-world evaluations of combined infection prevention and stewardship interventions are needed. Research should also explore the role of environmental and community reservoirs in sustaining hospital AMR. Finally, economic evaluations of early diagnostic implementation are warranted to determine their impact on reducing HAI-related mortality and healthcare costs.

## Conclusions

This meta-analysis highlights a clear shift in HAI etiology from predominantly Gram-positive organisms to a broader spectrum dominated by multidrug-resistant Gram-negative pathogens. The persistence and evolution of resistance, particularly in ESBL producers, carbapenem-resistant *A. baumannii*, and colistin-resistant Enterobacteriaceae, present urgent challenges to global health systems. Sustained investment in surveillance, prevention, stewardship, and novel therapeutics is essential to reverse these trends and safeguard the efficacy of existing antimicrobials.
