# Correction: Evolving Patterns of Etiological Profiles and Susceptibility Trends of Healthcare-Associated Infections Since Inception: A Meta-Analysis

**DOI:** 10.7759/cureus.c341

**Published:** 2025-10-01

**Authors:** Purvi S Khristi, Remya P A, Jeswin Chandrasekhar, Ami C Patel

**Affiliations:** 1 Microbiology, Dr. D.N. Desai Faculty of Medical Science and Research, Nadiad, IND; 2 Microbiology, Dharmsinh Desai University, Nadiad, IND; 3 Microbiology, Al-Azhar Medical College and Superspeciality Hospital, Thodupuzha, IND; 4 Pediatrics, Al-Azhar Medical College and Superspeciality Hospital, Thodupuzha, IND

This article has been revised to change the affiliation for Jeswin Chandrasekhar. His department has been changed from Microbiology to Pediatrics. Additionally, a second affiliation has been added for Purvi Khristi: Microbiology, Dharmsinh Desai University, Nadiad, India.

